# Relationship between theta/beta ratio and mind wandering in schizotypy

**DOI:** 10.1002/pchj.711

**Published:** 2023-12-17

**Authors:** Qin Zhang, Lu‐xia Jia, Ji‐fang Cui, Jun‐yan Ye, Jia‐li Liu, Wen‐hao Lai, Hai‐song Shi, Tian‐xiao Yang, Ya Wang, Raymond C. K. Chan

**Affiliations:** ^1^ Neuropsychology and Applied Cognitive Neuroscience Laboratory, CAS Key Laboratory of Mental Health Institute of Psychology Beijing China; ^2^ Department of Psychology University of Chinese Academy of Sciences Beijing China; ^3^ Shenzhen Children's Hospital Shenzhen China; ^4^ School of Education Guangzhou University Guangzhou China; ^5^ Research Center for Information and Statistics National Institute of Education Sciences Beijing China; ^6^ North China Electric Power University Beijing China; ^7^ School of Psychology Capital Normal University Beijing China

**Keywords:** attention, mind wandering, resting‐state electroencephalogram (EEG), schizotypal traits, theta/beta ratio

## Abstract

Negative association was found between the frontal theta/beta ratio and mind wandering in participants with high schizotypal traits, while no such association was found in participants with low schizotypal traits. These findings provide insights into the neural mechanism of mind wandering in individuals with high schizotypal traits.

## INTRODUCTION

Mind wandering (MW) is a pervasive phenomenon (occurring during approximately half of people's waking time) that refers to the appearance of task‐unrelated thoughts where attention is drawn away from the ongoing task (Killingsworth & Gilbert, 2010; Smallwood & Schooler, 2006). MW has adverse impacts upon cognition and life satisfaction (Blondé et al., 2022; Carciofo & Jiang, 2021; Zhang et al., 2022) but also plays a role in planning and creativity (Baird et al., 2011; Yamaoka & Yukawa, 2020a). Therefore, it is important to further study MW to get a deeper understanding of this phenomenon.

Schizophrenia patients have been found to mind wander more frequently than healthy controls, and the frequency of MW was found to be associated with positive symptoms (Iglesias‐Parro et al., 2020; Shin et al., 2015). Positive symptoms such as hallucinations and delusions are considered to be the main clinical symptoms in schizophrenia (Parnas, 2011; Tandon et al., 2013). Hallucinations and MW share some similarities, such as spontaneous, transient and relatively unconstrained nature; during these processes, individuals spontaneously disengage their attention from external events and are accompanied by excessive preoccupation with the inner world (Fazekas, 2021). It has been proposed that hallucinations could be considered to be intensified forms of MW that are produced by the same mechanisms (Fazekas, 2021). Therefore, studying MW has implications for psychopathology of schizophrenia.

Schizotypal traits reflect subclinical manifestations of psychotic symptoms in the general population (Debbané & Mohr, 2015). These traits include three dimensions, i.e., positive (cognitive‐perceptual), negative (interpersonal), and disorganized, which correspond to positive, negative, and general psychopathological symptoms in schizophrenia patients (Fonseca‐Pedrero et al., 2018; Raine, 1991; Raine et al., 1994). Individuals with high schizotypal traits are at risk for developing schizophrenia (Debbané et al., 2015; Lenzenweger, 2021). Studying these individuals can avoid confounding factors such as medication, course of disease and hospitalization in clinical samples, and contribute to the early identification of and intervention in schizophrenia (Barrantes‐Vidal et al., 2015).

A study revealed that individuals who reported more schizotypal traits were more likely to engage in MW (Yamaoka & Yukawa, 2020b). Studies also examined the association between MW and specific dimensions of schizotypal traits. For example, positive, disorganized and paranoid factors of schizotypal traits could predict the propensity for MW (Kane et al., 2016); adolescents with anhedonia (negative schizotypal traits) reported a higher MW frequency than controls (Webb et al., 2021). However, few studies to date have examined the neural correlates of MW in individuals with high schizotypal traits.

Resting‐state electroencephalograms (EEG) have been suggested to reflect intrinsic neurophysiological activities of the brain and are widely used to assess the brain function in healthy participants and clinical patients. The spectral power can be computed for different frequency bands, e.g., the slow wave activity (theta power, 4–7 Hz) and fast wave activity (beta power, 13–30 Hz). The ratio between theta power and beta power (theta/beta ratio, TBR) has been shown to be related to attentional control. For example, studies have reported a negative association between resting‐state TBR and attentional control in healthy adults (Angelidis et al., 2016; Putman et al., 2014). A study also showed that resting‐state frontal TBR was negatively correlated with attentional control in multiple sclerosis patients with mild cognitive impairment (Keune et al., 2017). The aforementioned studies provide support that the resting‐state TBR could be considered as a neurophysiological marker of attentional control in healthy and clinical groups. In addition, studies also examined MW during a breath‐counting task while the EEG data were recorded (van Son, De Blasio, et al., 2019; van Son, de Rover, et al., 2019). In those studies, healthy individuals were required to close their eyes and count their breaths, and press a button when they realized they were MW. Results revealed an increased TBR in the frontal region during MW compared to on‐task periods. These findings suggest a close relationship between TBR and MW.

However, whether the TBR would be associated with MW in individuals with high schizotypal traits remains unclear. The aim of the present study was to explore whether individuals with high schizotypal traits would show more MW than individuals with low schizotypal traits. Moreover, it also aimed to examine whether there would be a different association between resting‐state TBR and MW in individuals with high compared to low schizotypal traits.

## METHOD

Six‐hundred university students were recruited and completed the Schizotypal Personality Questionnaire (SPQ) online (Chen et al., 1997; Raine, 1991). Participants reporting SPQ total score within the top 10% of the entire sample (>40 in the present study) were considered as the high schizotypal group, whereas participants whose SPQ score was below average (< 25 in the present study) were considered as the low schizotypal group (Raine, 1991). The inclusion criteria were: no personal history of neurological or psychiatric disorders, no family history of psychiatric disorders, and no history of alcohol/substance abuse/dependence. As a result, 50 individuals with high schizotypal traits and 50 individuals with low schizotypal traits took part in the EEG study. Two high schizotypal individuals with excessive EEG artifacts were excluded from the final analysis. The study was approved by the ethics committee of the Institute of Psychology, Chinese Academy of Sciences. All participants provided written informed consent.

All participants completed the MW Questionnaire (Singer & Antrobus, 1963), which measures individual's MW frequency in daily life. Participants were required to respond from 1 (not at all) to 5 (very much) for 12 items. A higher score indicates a higher frequency of MW. This study adopted the Chinese version of the MWQ which shows good validity and reliability (Song & Tang, 2012). The internal consistency was 0.908 for this study.

As there might be EEG difference between eyes‐open (EO) and eyes‐closed (EC) conditions, participants were required to complete a 12‐minute resting‐state EEG recording session consisting of three 2 min EO blocks and three 2 min EC blocks in the following order: EO‐EC‐EO‐EC‐EO‐EC. Continuous EEG was recorded using a 64‐channel elastic cap, and the left mastoid was used as an online reference (Neuroscan system, Scan 4.5). Impedances of all electrodes were kept below 5 kΩ. EEG and EOG signals were digitized at a sampling rate of 1000 Hz and band‐pass filtered online between 0.05 and 100 Hz.

Data analyses were conducted using the custom MATLAB (Mathworks Inc., Natick, MA) scripts and functions from the EEGLAB toolbox. All EEG signals were first re‐referenced offline to the mean of left and right mastoids. Then a 1–30 Hz band‐pass (half amplitude) digital filter was applied to these signals using a basic finite impulse response (FIR) filter. Data were analyzed in two‐second segments. Independent component analysis (ICA) was performed in EEGLAB to exclude blink artifacts, we removed components (usually 1–4 components) (see Appendix A) with positive and negative waveforms distributed on both sides of the prefrontal area or concentrated in the prefrontal area, and examined whether eye‐blink‐related waveforms were diminished after removing the component(s). Then, trials exceeding a threshold of ±100 μV were excluded (if the peak was above +100 μV or below –100 μV, this trial would be removed) (see Appendix A). Participants with less than 85% valid trials were excluded from further analysis (two participants were excluded). Subsequently, a fast Fourier transformation (FFT) was performed, including a Hanning window that tapered data at the distal 10% of each epoch to avoid spurious elements of spectral power. The FFT yielded power spectral density for the theta (4–7 Hz) and beta (13–30 Hz) frequency band. Because of nonnormality, power spectral densities were log‐transformed before the calculation of TBR. The analysis involved spectral power density for frontal (Fz), central (Cz) and parietal (Pz) electrodes.

All statistical analyses were conducted with SPSS 26.0 (IBM). Group differences of age, duration of education, sex proportion, MW score and TBR were compared using independent sample *t*‐test and chi‐square test. To examine the potential association between TBR and MW, Pearson correlation was conducted between MW score and TBR for frontal (Fz), central (Cz) and parietal (Pz) electrodes for EC and EO conditions in the two groups separately. In addition, the differences in correlation coefficients between the two groups were compared.

## RESULTS

There was no significant difference in sex proportion (47.9% and 32% males), age (mean age 21.13 and 21.04), and duration of education (15.20 and 15.48 years) between the high schizotypal and low schizotypal groups (all *p*s > .1). There was no significant group difference of TBR in any electrode. However, the high schizotypal group (*M* = 41.96, *SD* = 8.14) showed significantly higher frequency of MW than the low schizotypal group (*M* = 32.88, *SD* = 7.32) (*p* < .001). The total MW score (ranged from 17 to 60) was normally distributed, with a skewness of 0.206 and kurtosis of −0.494.

**FIGURE 1:**
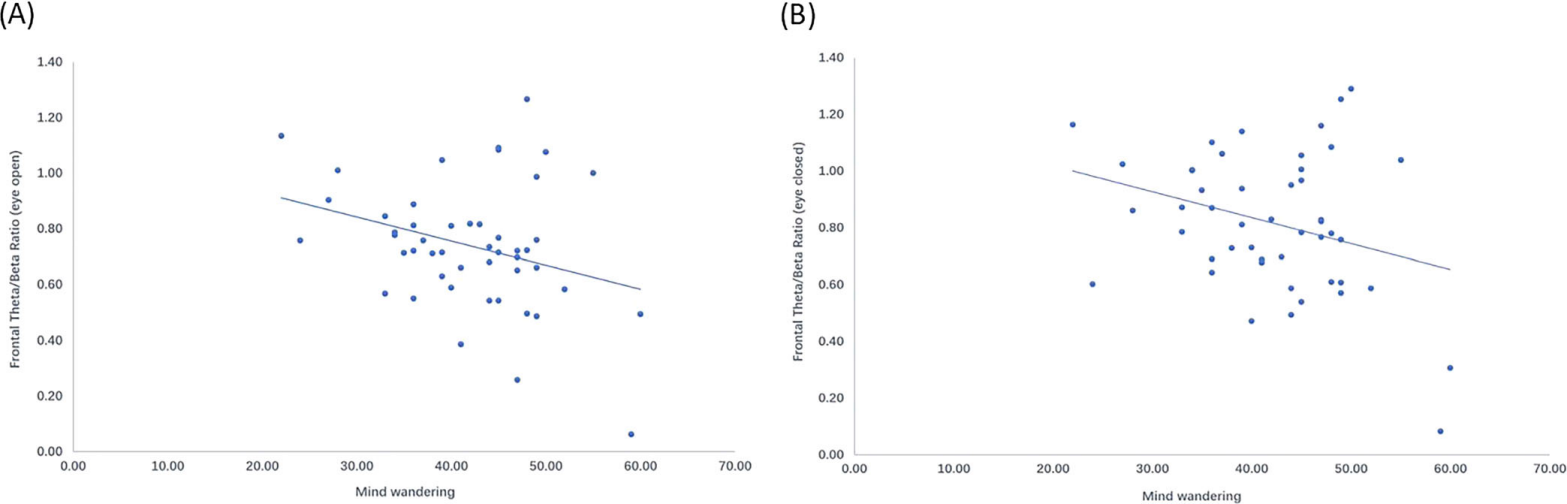
Scatterplots of the correlations between mind wandering and frontal theta/beta ratio in the high schizotypal group. (A) eyes‐open condition (*r* = −0.309, *p* = .033); (B) eyes‐closed condition (*r* = −0.302, *p* = .037).

There was no significant overall correlation between MW and TBR (*r* = −0.18−0.11, all *p*s > .05). For the high schizotypal group, frontal TBR showed a significantly negative correlation with MW in both conditions (EO: *r* = −0.309, *p* = .033; EC: *r* = −0.302, *p* = .037, see Figure 1 for scatterplots). However, for the low schizotypal group, frontal TBR showed no significant correlation with MW (EO: *r* = 0.040, *p* = .781; EC: *r* = 0.071, *p* = .626). Central and parietal TBR did not show significant correlations with MW in either group.

The strength of association between TBR at Fz and MW was significantly different between the high and low schizotypal groups for both EO and EC conditions. Significant group difference in the association between TBR at Cz for EO condition and MW, and between TBR at Pz for EC condition and MW was also found.

## DISCUSSION

The finding that the high schizotypal group showed a significantly higher frequency of MW compared with the low schizotypal group is in accordance with previous studies in patients with schizophrenia (Iglesias‐Parro et al., 2020; Shin et al., 2015) and individuals with high schizotypal traits (Kane et al., 2016; Webb et al., 2021; Yamaoka & Yukawa, 2020b; Zhang et al., 2022). These results suggest that frequent MW may be a risk factor for schizophrenia.

Though the high and low schizotypal groups did not show significant difference in TBR, different association patterns with MW were found in the two groups. Increased MW was associated with lower resting‐state frontal TBR in the high schizotypal group; however, no significant association between TBR and MW was found in the low schizotypal group. Dias da Silva et al. (2022) found that self‐report MW was negatively correlated with TBR during a visuomotor tracking task. It was consistent that self‐report MW was negatively associated with TBR in individuals with high schizotypal traits.

The different correlation patterns of MW between the high and low schizotypal groups and the finding of a higher frequency of MW in the high schizotypal group suggest that there may be important differences in the nature of MW between the two groups. It could be argued that differences in the correlation patterns between the two groups might be related to the abnormality of brain function in the high schizotypal group. Studies revealed that individuals with high schizotypal traits exhibited decreased executive control network functional connectivity (Shan et al., 2022).

In addition, research has shown that the connectivity of the ventromedial prefrontal cortex was positively correlated with self‐report MW in healthy controls but negatively correlated with self‐report MW in patients with schizophrenia, indicating decreased association between brain connectivity and MW in schizophrenia patients (Shin et al., 2015). Moreover, the resting‐state functional connectivity between medial prefrontal cortex and brain regions such as insula and intraparietal sulcus were positively associated with MW (measured by ecological momentary assessment) in the anhedonia (negative schizotypy) group but not the control group (Webb et al., 2021). The present study demonstrated that TBR was significantly associated with MW in individuals with high schizotypal traits but not those with low schizotypal traits. These findings suggest that there might be a differential relationship between MW and brain function in the schizophrenia spectrum. However, more studies are needed to examine the relationship between MW and TBR in individuals in this spectrum.

In conclusion, individuals with high schizotypal traits reported more MW than those with low schizotypal traits, and MW was negatively associated with frontal TBR in individuals with high schizotypal traits but not in those with low schizotypal traits.

## FUNDING INFORMATION

Ya Wang is funded by the National Science Foundation of China (32071062).

## CONFLICT OF INTEREST STATEMENT

None.

## ETHICS STATEMENT

The study was approved by the ethics committee of the Institute of Psychology, Chinese Academy of Sciences. All participants provided written informed consent.

## APPENDIX A

1. The number of components removed during ICA did not show significant difference (*t* = −1.40, *p* > .05) in the eyes‐open condition between the low schizotypal group (2.44 ± 0.76) and the high schizotypal group (2.71 ± 1.11). In the eyes‐closed condition, the number of components removed did not show significant difference (*t* = −0.31, *p* > .05) between the low schizotypal group (1.90 ± 0.68) and the high schizotypal group (1.85 ± 0.77) either.
2. The percentage of trials removed due to artifacts did not show significant difference (*t* = 0.08, *p* > .05) in the eyes‐open condition between the low schizotypal group (3.7% ± 1.8%) and the high schizotypal group (3.7% ± 2.0%). In the eyes‐closed condition, the percentage of trials removed due to artifacts did not show significant difference (*t* = 0.64, *p* > .05) between the low schizotypal group (3.4% ± 1.8%) and the high schizotypal group (3.2% ± 1.7%) either.
